# Health‐related quality of life in survivors of advanced melanoma treated with anti‐PD1‐based immune checkpoint inhibitors

**DOI:** 10.1002/cam4.5967

**Published:** 2023-04-29

**Authors:** E. L. Looman, P. F. Cheng, J. Lai‐Kwon, L. Morgan, M. Wakkee, R. Dummer, F. Dimitriou

**Affiliations:** ^1^ Department of Dermatology University Hospital of Zurich Zurich Switzerland; ^2^ Department of Dermatology Erasmus University Medical Center Rotterdam The Netherlands; ^3^ Faculty of Medicine University of Zurich Zurich Switzerland; ^4^ Department of Medical Oncology Peter MacCallum Cancer Centre Melbourne Australia

**Keywords:** immune checkpoint inhibitors, immunotherapy, melanoma, quality of life, survivorship

## Abstract

**Background:**

Immune checkpoint inhibitors (ICIs) have significantly improved survival in advanced melanoma but are associated with immune‐related adverse events (irAEs). This single center, cross‐sectional survey aimed to describe the long‐term symptom burden and impact on health‐related quality of life (HRQL) of advanced melanoma patients with sustained disease control following treatment with ICIs.

**Methods:**

Advanced melanoma patients (stage IIB, III or IV, AJCCv8), treated with anti‐PD1‐based ICIs, who were off‐treatment and had at least 6 months follow‐up from their last infusion with an ongoing response in the metastatic setting or no evidence of disease recurrence in the adjuvant setting. A paper‐based questionnaire, consisting of the EORTC QLQ‐C30, EORTC QLQ‐FA12, and the PRO‐CTCAE was administered.

**Results:**

Of 90 participants, 61 (68%) completed the questionnaire; 40 received single‐agent anti‐PD1, and 21 anti‐PD1/anti‐CTLA4. Thirty‐three (54%) were treated in the adjuvant setting. At the time of enrolment, 31 (51%) participants had active treatment for a previous irAE. Overall, 18/61 (30%) participants reported long‐term symptoms and trouble in physical and emotional functioning. Physical fatigue was common and interfered with daily activities (*n* = 12, 20%). In the PRO‐CTCAE questionnaire, muscle ache (*n* = 12, 20%) and joint ache (*n* = 9, 15%) were commonly reported. Despite this, participants reported overall good health (6.00, range 2.00–7.00) and reasonable level of HRQL (6.00, range 3.00–7.00).

**Discussion:**

Melanoma survivors experience long‐term symptoms in physical and psychosocial HRQL domains after ICI treatment. These results underline the importance to address existing gaps in survivorship care, implement these findings in clinical practice and increase awareness for long‐term symptoms in these patients.

## 1 INTRODUCTION

Immune checkpoint inhibitors (ICIs) have significantly improved the treatment outcome of advanced melanoma^1^, ^2^, ^3^ and are now also increasingly used in high‐risk, early‐stage melanoma as well.^4^ In patients with metastatic melanoma, combined treatment with ipilimumab/nivolumab results in durable responses in a growing number of patients, with a melanoma‐specific survival (MSS) of 55% (95% CI 50%–61%) after 7.5 years.^5^ Similarly, adjuvant treatment in patients with resected, stage III melanoma improves recurrence‐free survival (RFS),^6^, ^7^ whereas recent data from phase 3 clinical trials support their use even in resected stage II melanoma.^8^ These improvements underline that there is a growing number of patients with durable disease remission, which ultimately results in an emerging population of melanoma survivors.

When considering the risk–benefit ratio of ICI therapy, immune‐related adverse events (irAEs) need to be taken into consideration.^9^ Grade 3–4 irAEs have been reported to occur in 16% of patients treated with anti‐PD1 and 55% with ipilimumab/nivolumab. In 8% and 36% of the cases, respectively, irAEs may lead to treatment discontinuation.^10^, ^11^ Although irAEs are generally easily manageable, they can be also associated with long‐term functional impairment or even fatality.^12^, ^13^, ^14^ Chronic irAEs, namely those that persist at least 12 weeks after ICI therapy cessation, are estimated to occur in approximately 40% of patients treated with single‐agent anti‐PD1.^15^ However, due to their low‐frequency, chronic irAEs are frequently under‐reported and under‐recognized and may also have a significant impact on a person's overall health‐related quality of life (HRQL).

Patient‐reported outcomes (PROs) could complement the report of chronic irAEs and could be used to determine their onset and frequency from patients' perspective. Previous studies collecting patient‐reported outcome measures (PROMs) in people with advanced melanoma have demonstrated multiple symptoms including arthralgias, muscle ache and generalized pain or discomfort.^16^, ^17^, ^18^ Somatic symptoms, such as fatigue, as well as psychological concerns, such as anxiety or depression are commonly reported among melanoma survivors, although there seems to be a high heterogeneity with regard to their frequency.^16^, ^17^, ^19^, ^20^ Implementation of these data in patient care is critical to optimize treatment and management of this growing survivor population.

In the present study, we aim to describe the symptom burden and impairments in HRQL in melanoma patients treated either in the adjuvant or in the metastatic setting, with sustained disease control and without disease recurrence for at least 6 months after the last infusion of anti‐PD1‐based ICIs using PROMs. We further analyze these results according to treatment type, in order to unravel the long‐term symptoms of patients that discontinued treatment due to irAEs.

## 2 METHODS

This is a single‐institution, cross‐sectional study with both retrospective chart review and prospective data collection. The primary objective of the study was to describe the symptom burden and HRQL in patients alive and off‐treatment, with sustained disease control at least 6 months after the last dose of ICIs. The secondary objectives were to assess the differences in the symptom burden and HRQL according to ICI treatment type, age and sex. Ethics approval and consent to participate was obtained from the local ethics committee (KEK Zürich, BASEC‐Nr. 2022–00448). Patients' enrollment followed upon collection of written informed consent.

### 2.1 Eligibility criteria

Patients diagnosed with advanced melanoma (stage IIB, III or IV, American Joint Committee on Cancer [AJCC] version 8) and treated either with single‐agent anti‐PD1 or anti‐PD1 in combination with anti‐CTLA4 (ipilimumab/nivolumab) were included. Of note, there was one patient with locally advanced mucosal melanoma (stage IIB) that was treated with single‐agent anti‐PD1. Patients were treated according to the proposed recommendations for the management of advanced melanoma and upon interdisciplinary tumorboard discussion; patients in the adjuvant setting were treated with single‐agent anti‐PD1, while patients in the metastatic setting received either single‐agent anti‐PD1 or anti‐PD1/anti‐CTLA4.^21^ In order to accurately evaluate the symptom burden in the population of melanoma survivors, eligible patients had to be off‐treatment and have a follow‐up of at least 6 months since their last infusion. Eligible participants were alive with ongoing disease remission after achieving an objective response [complete or partial response, as well as stable disease for at least 6 months evaluated according to the RECIST 1.1 criteria^22^] in the metastatic setting or were alive with no evidence of disease recurrence in the adjuvant setting. Other inclusion criteria included ability to speak and read German or English and ability to provide an informed consent. Patients that received any subsequent systemic anti‐cancer treatment after the initiation of ICIs, as well as patients with disease recurrence (adjuvant setting) or disease progression (metastatic setting), were excluded.

### 2.2 Participant recruitment

Patients' enrollment was performed between January 2022 and July 2022 (data cut‐off). Institutional database for medical records was reviewed to identify eligible participants. Participants identified as eligible were contacted by phone or approached in the clinic.

### 2.3 Data collection

Baseline patient characteristics, including sex, age, ECOG performance status (PS), presence of autoimmune diseases and active immunosuppression, were retrieved from the available medical records. Disease and treatment characteristics, as well as type and grade of irAEs and reason for treatment discontinuation, were retrospectively collected. In patients that discontinued treatment due to irAEs, duration of symptoms after ICI discontinuation, and onset of new toxicities while off‐treatment were documented. In patients that discontinued treatment for reasons other than irAEs (e.g., completion of treatment, elective discontinuation), onset of new toxicities while off‐treatment and duration of toxicities were also collected. The severity of irAEs was graded according to the Common Terminology Criteria for Adverse Events version 5 (CTCAEv5).

### 2.4 Questionnaire

Study participants received a study‐specific, paper‐based questionnaire. The questionnaire consisted of the validated questions provided by the European Organization for Research and Treatment of Cancer (EORTC) and included the EORTC QLQ‐FA12 questionnaire, measuring the cancer‐related fatigue, and the EORTC QLQ‐C30 questionnaire, developed to assess the quality of life of cancer patients.^23^, ^24^, ^25^ In addition, we selected a subset of questions from the PROs version of the CTCAE (PRO‐CTCAE) questionnaire that assesses the symptom burden and impact of 78 toxicities adapted by the CTCAE (available in Data S1). The selected items were sought to assess symptoms from long‐term toxicities, including endocrinopathies, as well as rheumatological, cutaneous and neurological toxicities.^26^, ^27^ The final questionnaire consisted of 79 items that were used in their original format and a final free‐text question to report any other symptoms. The items could be rated on a 4‐ to 5‐point scale (QLQ‐FA12 and QLQ‐C30: 1 = not at all, 2 = a little, 3 = quite a bit, 4 = very much; PRO‐CTCAE: 1 = none/not at all/never, 2 = mild/a little bit/rarely, 3 = moderate/somewhat/occasionally, 4 = severe/quite a bit/frequently, 5 = very severe/very much/almost constantly). The items regarding overall health and overall HRQL in the QLQ‐C30 questionnaire could be rated on a 7‐point scale (1 = very poor, 7 = excellent). Patients could complete the paper questionnaire on the day of enrolment or return it later via a reply‐paid envelope or per e‐mail. Proxies were permitted for patients that could not complete the questionnaire by themselves. Patients who did not return the questionnaire after 4 weeks received a reminder over the phone.

### 2.5 Statistical analysis

Demographic and clinical characteristics were summarized using descriptive statistics, including mean (range) and frequency (percentage). For the statistical analysis, results from the EORTC questionnaire were: not at all/very little (1–2) and quite a bit/very much (3–4). For the PRO‐CTCAE questionnaire, results were collapsed: none/mild (1–2), moderate (3) and severe/very severe (4–5). Subgroup analysis according to treatment agent, sex and age, with a cut‐off of 65 years, in accordance with melanoma phase 3 clinical trials (CheckMate 067, Columbus, KEYNOTE‐006), was performed. Differences between the treatment groups were compared using Fisher's exact test or Pearson's chi squared test for discrete variables (e.g., sex), and Wilcoxon rank sum test for continuous variables (e.g., age). All analyses were conducted using statistical language R version 4.2.1 (R foundation, USA). A two‐sided *p*‐value <0.05 was considered statistically significant. The questionnaire responses were evaluated using descriptive analyses.

## 3 RESULTS

**FIGURE 1:**
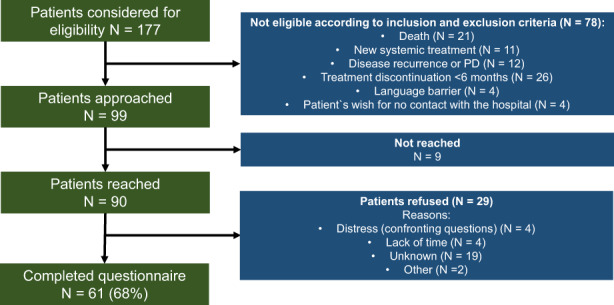
Study flowchart. ICIs, immune checkpoint inhibitors; *N*, number; PD, progressive disease.

Of the 177 patients considered for eligibility, 99 patients fulfilled the eligibility criteria and were subsequently approached for participation in the study. Of the 90 patients reached by phone, 61 patients completed the questionnaire, corresponding to a survey response rate of 68% (Figure 1). Twenty‐nine patients refused to participate. Non‐responders had a median age of 67 years (range 21–88 years), were more likely males (55%) and were mostly treated with anti‐PD1 (83%). Reasons for non‐response after receiving the questionnaire included lack of time (*N* = 4, 14%) or distress due to confronting questions (*N* = 4, 14%).

### 3.1 Participant characteristics

Baseline participant characteristics are summarized in Table 1. Of the 61 participants included, median age at time of study enrollment was 63 years (range 30–84 years), 38 (62%) were males and 46 (75%) were diagnosed with cutaneous melanoma. Three (5%) participants had pre‐existing autoimmune disease at treatment initiation and three (5%) participants received immunosuppressive treatment at the time of the study enrolment. Overall, 21 (34%) patients were treated with ipilimumab/nivolumab and 40 (66%) with single‐agent anti‐PD1. Thirty‐three (54%) patients were treated in the adjuvant and 28 (46%) in the metastatic setting. Median time on treatment was 12 months (range 0–42 months). Median time from treatment discontinuation to study enrollment was 26 months (range 6–73 months) and reasons for treatment discontinuation were completion of treatment course (*n* = 31, 51%) or irAEs (*n* = 18, 30%). The median time of follow‐up (FU) after treatment discontinuation was 26 months (range 6–73 months).

**TABLE 1:** Baseline characteristics.

	Overall	Anti‐PD1	Anti‐PD1 and Anti‐CTLA4	*p*‐value^a^
	*N* = 61	*N* = 40	*N* = 21	
Age at time of study enrollment (median, range)	63 (30–84)	64 (30–84)	63 (34–84)	0.5
Sex (%)				0.029
Female	23 (38%)	19 (48%)	4 (19%)	
Male	38 (62%)	21 (52%)	17 (81%)	
Melanoma subtype (%)				0.3
Acral lentiginous	2 (3%)	1 (3%)	1 (5%)	
Cutaneous	46 (75%)	33 (82%)	13 (62%)	
Mucosal	3 (5%)	1 (3%)	2 (10%)	
Unknown primary	10 (16%)	5 (12%)	5 (24%)	
Prior treatment (%)				0.7
None	47 (77%)	31 (78%)	16 (76%)	
BRAF/MEK Inhibitors	1 (2%)	0	1 (5%)	
ICIs	9 (15%)	5 (12%)	4 (19%)	
ICIs and BRAF/MEK Inhibitors	2 (3%)	2 (5%)	0	
ICIs and T‐VEC	1 (2%)	1 (3%)	0	
Other	1 (2%)	1 (3%)	0	
Pre‐existing autoimmunity at treatment initiation (%)	3 (5%)	2 (5%)	1 (5%)	>0.9
Presence of immunosuppressive treatment at treatment initiation (%)	3 (5%)	2 (5%)	1 (5%)	>0.9
Stage at treatment initiation (%)				<0.001
IIB	1 (2%)	1 (3%)	0	
IIIA	3 (5%)	3 (8%)	0	
IIIB	16 (26%)	16 (40%)	0	
IIIC	12 (20%)	12 (30%)	0	
IV	29 (48%)	8 (20%)	21 (100%)	
Treatment setting (%)				<0.001
Adjuvant	33 (54%)	33 (82%)	0 (0%)	
Metastatic	28 (46%)	7 (18%)	21 (100%)	
Median Time on treatment (months) (median, range)	12 (0–42)	12 (0–42)	15 (1–37)	0.3
Number of anti‐PD1 cycles (median, range)	18 (3–64)	18 (3–64)	18 (3–48)	0.9
Total number of anti‐PD1/anti‐CTLA4 cycles (%)				>0.9
1	3 (14%)	—	3 (14%)	
2	5 (24%)	—	5 (24%)	
3–4	13 (62%)	—	13 (62%)	
BOR (%)				0.5
CR	21 (75%)	5 (71%)	16 (76%)	
PR	6 (21%)	1 (14%)	5 (24%)	
SD	1 (4%)	1 (14%)	0	
Unknown	33	33	0	
Brain metastases at treatment start (%)				<0.001
Yes	11 (18%)	2 (5%)	9 (43%)	
Liver metastases at treatment start (%)				0.042
Yes	7 (11%)	2 (5%)	5 (24%)	
Bone metastases at treatment start (%)				0.2
Yes	6 (10%)	2 (5%)	4 (19%)	
ECOG PS at treatment start (%)				0.3
0	56 (92%)	38 (95%)	18 (86%)	
≥4	5 (8%)	2 (5%)	3 (14%)	
Reason for treatment discontinuation (%)				0.012
Adverse event	18 (30%)	8 (20%)	10 (48%)	
Complete/Partial response	10 (16%)	7 (18%)	3 (14%)	
Completed treatment	31 (51%)	25 (62%)	6 (29%)	
Patient's or Investigator's decision	2 (3%)	0	2 (10%)	
Time since treatment discontinuation (months) (median, range)	26 (4–73)	28 (6–73)	22 (4–58)	0.6

Abbreviations: BOR, best overall response; CR, complete response; ICI, immune checkpoint inhibitor; IrAE, immune‐related adverse event; PR, partial response; SD, stable disease.

^a^ Wilcoxon rank sum test; Pearson's Chi‐squared test; Fisher's exact test.

### 3.2 Occurrence of immune‐related adverse events

irAEs of any grade occurred in 56 (92%) patients during treatment; 35 (88%) treated with anti‐PD1 and 21 (100%) with ipilimumab/nivolumab. Twenty‐one patients (34%) of the overall population experienced grade 3–4 irAEs; 17 (81%) were treated with ipilimumab/nivolumab and four (10%) with single‐agent anti‐PD1. In patients treated with ipilimumab/nivolumab, the most common irAEs included dermatitis (52%), hepatitis (52%) and colitis (43%), whereas anti‐PD1‐related irAEs included thyroiditis (30%), hepatitis (23%), and dermatitis (23%). In 18 (30%) patients, irAEs led to treatment discontinuation and common irAEs included pneumonitis (17%), arthritis (17%), and nephritis (11%) (Table S1 *, available online*). At the time of enrolment, 31 (51%) patients had either ongoing symptoms or required active treatment for a previous irAE. Endocrinopathies were commonly associated with ongoing active treatment and included thyroiditis/hypothyroidism (20%), adrenal insufficiency (7%), hypophysitis (5%), and type 1 diabetes (2%). Notably, patients with ongoing endocrine irAEs were more likely to report fatigue in the PRO‐CTCAE (*p* = 0.01) and EORTC QLQ‐FA12 questionnaires (Table S2 *, available online*). Table 2 summarizes the irAEs characteristics.

**TABLE 2:** Summary and frequency of immune‐related adverse events (irAEs) during treatment and those requiring active treatment at the time of study enrollment (chronic irAEs).

	IrAEs during treatment			Grade ≥3 irAEs during treatment			Chronic irAEs	
	Overall (*N*, %)	Anti‐PD1 (*N*, %)	Anti‐PD1 and Anti‐CTLA4 (*N*, %)	Overall (*N*, %)	Anti‐PD1 (*N*, %)	Anti‐PD1 and Anti‐CTLA4 (*N*, %)	Overall (*N*, %)	Type of ongoing treatment
Adrenal Insufficiency	4 (7%)	2 (5%)	2 (10%)	—	—	—	4 (7%)	Hydrocortisone
Arthritis	8 (13%)	4 (10%)	4 (19%)	4 (7%)	1 (3%)	3 (14%)	3 (5%)	Prednisone, anti‐TNFα
Bronchitis	1 (2%)	1 (3%)	0	—	—	—	0	—
Colitis	14 (23%)	5 (13%)	9 (43%)	3 (5%)	1 (3%)	2 (10%)	2 (3%)	Anti‐α4β7‐integrin, anti‐TNFα
Dermatitis/Skin Rash	20 (33%)	9 (23%)	11 (52%)	2 (3%)	—	2 (10%)	2 (3%)	Prednisone, local corticosteroids
Type 1 Diabetes	1 (2%)	0	1 (5%)	1 (2%)	—	1 (5%)	1 (2%)	Insulin
Gastritis/Duodenitis/Enteritis	4 (7%)	2 (5%)	2 (10%)	2 (3%)	1 (3%)	1 (5%)	1 (2%)	Prednisone
Hepatitis	20 (33%)	9 (23%)	11 (52%)	7 (12%)	1 (3%)	6 (29%)	1 (2%)	Prednisone
Hypophysitis	6 (10%)	1 (3%)	5 (24%)	1 (2%)	—	1 (5%)	3 (5%)	Hydrocortisone
Keratoconjunctivitis Sicca	3 (5%)	2 (5%)	1 (5%)	—	—	—	0	—
Myositis	1 (2%)	0	1 (5%)	1 (2%)	—	1 (5%)	0	—
Nephritis	3 (5%)	1 (3%)	2 (10%)	2 (3%)	—	2 (10%)	1 (2%)	Prednisone
Neutropenia	1 (2%)	0	1 (5%)	1 (2%)	—	1 (5%)	0	—
Pancreatitis	12 (20%)	4 (10%)	8 (38%)	1 (2%)	—	1 (5%)	0	—
Pneumonitis	9 (15%)	6 (15%)	3 (14%)	—	—	—	1 (2%)	Prednisone, Hydroxychloroquine
Polyneuropathy/Neuritis/Encephalitis	3 (5%)	0	3 (14%)	1 (2%)	—	1 (5%)	0	—
Sarcoid‐like reaction	9 (15%)	4 (10%)	5 (24%)	1 (2%)	1 (3%)	—	0	—
Perimyocarditis	1 (2%)	1 (3%)	0	—	—	—	0	—
Thrombocytopenia	1 (2%)	1 (3%)	0	—	—	—	0	—
Thyroiditis (Hypothyroidism)	19 (31%)	12 (30%)	7 (33%)	—	—	—	12 (20%)	Levothyroxin
Troponin increase	3 (5%)	2 (5%)	1 (5%)	—	—	—	0	—
Vitiligo	5 (9%)	5 (13%)	0	—	—	—	0	—
Xerostomia	3 (5%)	3 (7%)	0	—	—	—	0	—

*Note*: Severity grading according to Common Terminology Criteria for Adverse Events [CTCAE], version 5.

Abbreviations: *N*, number; irAEs, immune‐related adverse events.

### 3.3 Symptom burden and impact on quality of life (HRQL)

In the questions investigating the overall health and HRQL in the EORTC QLQ‐C30 questionnaire, patients rated their overall health during the past week with 6.00 (range, 2.00–7.00) and their overall HRQL during the past week with 6.00 (range, 3.00–7.00) (Table 3). Overall, patients reported difficulties in physical functioning, such as trouble taking a long walk (18%) and limitation in pursuing hobbies and other leisure activities (21%), emotional functioning, expressing mostly worriedness (25%), and other symptoms and problems associated with fatigue, such as trouble sleeping (21%), tiredness (26%), and need of rest (25%). The rates of difficulties in physical, cognitive, emotional and social functioning, as well as in symptoms and problems, varied between anti‐PD1 and ipilimumab/nivolumab. Patients treated with ipilimumab/nivolumab were more likely to report difficulties in physical and social functioning. Difficulties in emotional functioning, such as worriedness, were common in both treatments (23% and 29%), but patients treated with anti‐PD1 were more likely to report tension (23% vs. 5%, *p* = 0.01). Symptoms and problems associated with tiredness and fatigue were common between the two treatments. In all, patients treated with ipilimumab/nivolumab rated their HRQL with a lower overall score compared to those treated with anti‐PD1 (5.10 and 5.72, respectively, *p* = 0.04).

**TABLE 3:** Levels of functioning (EORTC QLQ‐C30) in the overall study population and according to the treatment type.

	Overall	Anti‐PD1	Anti‐PD1 and Anti‐CTLA4	*p*‐value^a^
	*N* = 61	*N* = 40	*N* = 21	
Physical functioning (*n*, %)				
Do you have any trouble doing strenuous activities, like carrying a heavy shopping bag or a suitcase?	9 (15%)	4 (10%)	5 (24%)	0.4
Do you have any trouble taking a long walk?	11 (18%)	6 (15%)	5 (24%)	0.4
Do you have any trouble taking a short walk outside of the house?	1 (2%)	1 (3%)	0	0.3
Do you need to stay in bed or a chair during the day?	7 (11%)	4 (10%)	3 (14%)	0.4
Were you limited in doing either your work or other daily activities?	8 (13%)	5 (13%)	3 (14%)	0.9
Were you limited in pursuing your hobbies or other leisure time activities?	13 (21%)	7 (18%)	6 (29%)	0.2
Symptoms/Problems (*n*, %)				
Were you short of breath?	8 (13%)	7 (18%)	1 (5%)	0.5
Have you had pain?	9 (15%)	7 (18%)	2 (10%)	0.9
Did you need to rest?	15 (25%)	11 (28%)	4 (19%)	0.6
Have you had trouble sleeping?	13 (21%)	8 (20%)	5 (24%)	0.11
Have you felt weak?	10 (16%)	5 (13%)	5 (24%)	0.6
Have you lacked appetite?	3 (5%)	3 (8%)	0	0.7
Have you felt nauseated?	4 (7%)	4 (10%)	0	0.7
Have you vomited?	2 (3%)	2 (5%)	0	>0.9
Have you been constipated?	4 (7%)	2 (5%)	2 (10%)	>0.9
Have you had diarrhea?	3 (5%)	3 (8%)	0	0.3
Were you tired?	16 (26%)	11 (28%)	5 (24%)	0.8
Did pain interfere with your daily activities?	5 (8%)	4 (10%)	1 (5%)	>0.9
Cognitive functioning (*n*, %)				
Have you had difficulty in concentrating on things? like reading a newspaper or watching television?	9 (15%)	6 (15%)	3 (14%)	0.4
Have you had difficulty remembering things?	8 (13%)	5 (13%)	3 (14%)	>0.9
Emotional functioning (*n*, %)				
Did you feel tense?	10 (16%)	9 (23%)	1 (5%)	0.01
Did you worry?	15 (25%)	9 (23%)	6 (29%)	0.7
Did you feel irritable?	9 (15%)	6 (15%)	3 (14%)	0.7
Did you feel depressed?	6 (10%)	3 (8%)	3 (14%)	0.5
Social functioning (*n*, %)				
Has your physical condition or medical treatment interfered with your family life?	10 (16%)	6 (15%)	4 (19%)	>0.9
Has your physical condition or medical treatment interfered with your social activities?	9 (15%)	3 (8%)	6 (29%)	0.2
Has your physical condition or medical treatment caused you financial difficulties?	5 (8%)	3 (8%)	2 (10%)	0.5
How would you rate your overall health during the past week? (median, range)	6.00 (2.00–7.00)	6.00 (3.00–7.00)	5.00 (2.00–7.00)	0.13
How would you rate your overall quality of life during the past week? (median, range)	6.00 (3.00–7.00)	6.00 (4.00–7.00)	5.00 (3.00–7.00)	0.04

*Note*: Results are collapsed: quite a bit/very much (3–4). Only these results are shown.

^a^ Pearson's Chi‐squared test; Fisher's exact test.

Fatigue, and particularly physical fatigue, was a common symptom with frequency that was overall similar across the different questionnaires. In the QLQ‐FA12 questionnaire, 46/61 (75%) patients reported some extent of fatigue, and the corresponding frequencies were 36/61 (59%) in the QLQ‐C30 and 37/61 (61%) in the PRO‐CTCAE questionnaires. In the overall population, 14 (23%) patients reported lack of energy, 13 (21%) exhaustion and 14 (23%) sleepiness during the day. Other symptoms included feeling slowed down (*n* = 16, 26%) and having trouble getting things started (*n* = 15, 25%). The level of fatigue interfered with daily activities in 12 (20%) patients. The level of fatigue did not differ significantly in patients who received ipilimumab/nivolumab than in those who received single‐agent anti‐PD1. Nevertheless, overall fatigue was more prominent in the former than the latter. Notably, although physical fatigue was common in both treatments, emotional fatigue was higher in patients treated with ipilimumab/nivolumab (*n* = 21, 43%) and cognitive fatigue in patients treated with single‐agent anti‐PD1 (*n* = 8, 20%). Other differences were noted in the proportion of patients that reported exhaustion (29% vs. 18%, *p* = 0.3) and interference of tiredness with daily activities (24% vs 18%, *p* = 0.7). Results from the Cancer Related Fatigue Module (EORTC QLQ‐FA12) are summarized in Table 4.

**TABLE 4:** The Cancer Related Fatigue Module (EORTC QLQ‐FA12) in the overall study population and according to the treatment type.

	Overall	Anti‐PD1	Anti‐PD1 and Anti‐CTLA4	*p*‐value^a^
	*N* = 61	*N* = 40	*N* = 21	
Have you lacked energy?	14 (23%)	9 (22%)	5 (24%)	>0.9
have you felt exhausted?	13 (21%)	7 (18%)	6 (29%)	0.3
Have you felt slowed down?	16 (26%)	9 (22%)	7 (33%)	0.4
Did you feel sleepy during the day?	14 (23%)	9 (22%)	5 (24%)	>0.9
Did you have trouble getting things started?	15 (25%)	9 (22%)	6 (29%)	0.6
Did you feel discouraged?	8 (13%)	4 (10%)	4 (19%)	0.4
Did you feel helpless?	6 (10%)	4 (10%)	2 (10%)	>0.9
Did you feel frustrated?	7 (11%)	4 (10%)	3 (14%)	0.7
Did you have trouble thinking clearly?	6 (10%)	5 (12%)	1 (5%)	0.7
Did you feel confused?	3 (5%)	3 (8%)	0	0.5
Did tiredness interfere with your daily activities?	12 (20%)	7 (18%)	5 (24%)	0.7
Did you feel that your tiredness is (was) not understood by the people who are close to you?	1 (2%)	1 (3%)	0	>0.9

*Note*: Results are collapsed: quite a bit/very much (3–4). Only these results are shown.

^a^ Pearson's Chi‐squared test; Fisher's exact test.

### 3.4 Patient‐reported outcomes for symptoms on the PRO‐CTCAE

The most commonly reported symptoms on the PRO‐CTCAE questionnaire were muscle ache (*n* = 12, 20%), joint ache (*n* = 9, 15%), and generalized pain (*n* = 8, 13%), whereas sadness (*n* = 12, 20%) and depression (*n* = 7, 11%) were also occasionally reported (Table 5). These symptoms largely interfered with daily activity, as well. Severe symptoms included dry skin (*n* = 12, 20%), joint ache (*n* = 11, 18%), and decreased sexual interest (*n* = 11, 18%). Although there were no significant differences with regards to their frequency and severity between the two treatment regimens, patients treated with ipilimumab/nivolumab were more likely to report severe skin itchiness (24% vs. 8%), joint ache (24% vs. 15%), fatigue (24% vs. 12%), and decreased sexual interest (24% vs. 15%). In contrast, severe muscle ache was more prominent in single‐agent anti‐PD1 (5% vs. 20%). Similarly, rates of difficulties that interfered with physical activities did not differ between ipilimumab/nivolumab and single‐agent anti‐PD1, but generalized pain (10% vs. 15%) and muscle ache (5% vs. 15%) were commonly noted in single‐agent anti‐PD1 and joint ache (19% vs. 10%) in ipilimumab/nivolumab.

**TABLE 5:** Symptom burden (PRO‐CTCAE) for selected conditions in the overall study population and according to the treatment type.

	Overall	Anti‐PD1	Anti‐PD1 and Anti‐CTLA4	*p*‐value^a^
	*N* = 61	*N* = 40	*N* = 21	
Frequency				

	Frequently/almost constantly	Occasionally	Frequently/almost constantly	Occasionally	Frequently/almost constantly	Occasionally	
Pain	8 (13%)	8 (13%)	6 (15%)	4 (10%)	2 (10%)	4 (19%)	0.7
Headache	1 (2%)	7 (11%)	1 (3%)	5 (12%)	0	2 (10%)	>0.9
Muscle ache	12 (20%)	7 (11%)	9 (22%)	5 (12%)	3 (14%)	2 (10%)	0.8
Joint ache	9 (15%)	11 (18%)	6 (15%)	4 (10%)	3 (14%)	7 (33%)	0.08
Anxiety	1 (2%)	5 (9%)	1 (3%)	2 (5%)	0	3 (14%)	0.6
Depression	1 (2%)	7 (11%)	1 (3%)	3 (8%)	0	4 (19%)	0.4
Sadness	1 (2%)	12 (20%)	1 (3%)	5 (12%)	0	7 (33%)	0.08
Severity							

	Severe/very severe	Moderate	Severe/very severe	Moderate	Severe/very severe	Moderate	
Dry mouth	9 (15%)	5 (8%)	7 (18%)	4 (10%)	2 (10%)	1 (5%)	0.6
Dry skin	12 (20%)	14 (23%)	8 (20%)	8 (20%)	4 (19%)	6 (29%)	0.7
Itchy skin	8 (13%)	8 (13%)	3 (8%)	5 (12%)	5 (24%)	3 (14%)	0.2
Pain	8 (13%)	5 (8%)	5 (12%)	5 (12%)	3 (14%)	0	0.3
Headache	1 (2%)	4 (7%)	1 (3%)	3 (8%)	0	1 (5%)	>0.9
Muscle ache	9 (15%)	11 (18%)	8 (20%)	7 (18%)	1 (5%)	4 (19%)	0.3
Joint ache	11 (18%)	8 (13%)	6 (15%)	5 (12%)	5 (24%)	3 (14%)	0.7
Insomnia	9 (15%)	12 (20%)	6 (15%)	5 (12%)	3 (14%)	7 (33%)	0.1
Fatigue	10 (16%)	9 (15%)	5 (12%)	6 (15%)	5 (24%)	3 (14%)	0.5
Anxiety	1 (2%)	4 (7%)	1 (3%)	2 (5%)	0	2 (10%)	0.7
Depression	3 (5%)	5 (8%)	1 (3%)	2 (5%)	2 (10%)	3 (14%)	0.2
Sadness	4 (7%)	5 (8%)	2 (5%)	3 (8%)	2 (10%)	2 (10%)	0.7
Decreased sexual interest	11 (18%)	5 (8%)	6 (15%)	3 (8%)	5 (24%)	2 (10%)	0.6
Interference with daily activity							

	Quite a bit/very much	Somewhat	Quite a bit/very much	Somewhat	Quite a bit/very much	Somewhat	
Pain	8 (13%)	0	6 (15%)	0	2 (10%)	0	0.7
Headache	0	2 (3%)	0	2 (5%)	0	0	0.5
Muscle ache	7 (11%)	5 (8%)	6 (15%)	3 (8%)	1 (5%)	2 (10%)	0.6
Joint ache	8 (13%)	7 (11%)	4 (10%)	4 (10%)	4 (19%)	3 (14%)	0.5
Insomnia	9 (15%)	9 (15%)	6 (15%)	3 (8%)	3 (14%)	6 (29%)	0.1
Fatigue	7 (11%)	6 (10%)	5 (12%)	2 (5%)	2 (10%)	4 (19%)	0.3
Anxiety	1 (2%)	2 (3%)	1 (3%)	1 (3%)	0	1 (5%)	>0.9
Depression	2 (3%)	3 (5%)	1 (3%)	1 (3%)	1 (5%)	2 (10%)	0.4
Sadness	3 (5%)	2 (3%)	2 (5%)	1 (3%)	1 (5%)	1 (5%)	>0.9

*Note*: Results are collapsed: none/mild (1–2), moderate/occasionally/somewhat (3) and frequently/almost constantly, severe/very severe, quite a bit/very much (4–5).

^a^ Pearson's Chi‐squared test; Fisher's exact test.

### 3.5 Patient‐reported outcomes for age and sex

The results in the overall health and HRQL in the EORTC QLQ‐C30 and EORTC QLQ‐FA12 questionnaires did not differ when analyzed according to age and sex (results not shown). On the PRO‐CTCAE questionnaire, joint ache was more frequent in women (*n* = 7, 30%) than in men (*n* = 2, 5%) (*p* = 0.02, Table 6). Additionally, women were more likely to report severe dry skin (35% vs. 11%, *p* = 0.04) and muscle ache (30% vs. 5%, *p* = 0.02) than men. Nevertheless, symptoms were not significantly different when analyzed according to age, although muscle ache significantly interfered with physical activities in patients ≥65 years (*p* = 0.04).

**TABLE 6:** Symptom burden (PRO‐CTCAE) for selected conditions in the overall study population and according to sex and age.

	Overall	Females	Males	*p*‐value^a^	Age < 65 years	Age ≥ 65 years	*p*‐value^a^
	*N* = 61	*N* = 23	*N* = 38		*N* = 33	*N* = 28	
	Frequently/most constantly				Frequently/most constantly		
Muscle ache	12 (20%)	7 (30%)	5 (13%)	0.2	5 (15%)	7 (25%)	0.3
Joint ache	9 (15%)	7 (30%)	2 (5%)	0.02	5 (15%)	4 (14%)	>0.9
	Severe/very severe				Severe/very severe		
Dry skin	12 (20%)	8 (35%)	4 (11%)	0.04	7 (21%)	5 (18%)	0.7
Muscle ache	9 (15%)	7 (30%)	2 (5%)	0.02	3 (9%)	6 (21%)	0.3
Joint ache	11 (18%)	7 (30%)	4 (11%)	0.08	5 (15%)	6 (21%)	0.5
	Interference with daily activity: quite a bit/very much				Interference with daily activity: quite a bit/very much		
Muscle ache	7 (11%)	5 (22%)	2 (5%)	0.09	1 (3%)	6 (21%)	0.04
Joint ache	8 (13%)	5 (22%)	3 (8%)	0.14	3 (9%)	5 (18%)	0.5

*Note*: Results are collapsed: none/mild (1–2) moderate/occasionally/somewhat (3) and frequently/almost constantly, severe/very severe, quite a bit/very much (4–5).

^a^ Pearson's Chi‐squared test; Fisher's exact test.

## 4 DISCUSSION

Anti‐PD1‐based treatment is currently standard‐of‐care in patients with advanced melanoma and can result in durable disease remission in a subgroup of patients. This cohort study showed that patients with sustained disease control or absence of disease recurrence for at least 6 months after the last infusion of anti‐PD1‐based ICIs, experience long‐term symptoms, albeit with an overall good health and reasonable level of HRQL. Overall, participants reported difficulties in physical and emotional functioning, while physical fatigue was a common symptom in the overall study population. Other symptoms included muscle ache and joint ache, whereas generalized pain commonly interfered with daily activities. Although there were no substantial differences between the two treatment modalities, patients treated with ipilimumab/nivolumab had a global lower score in their HRQL compared to single‐agent anti‐PD1. This was also in line with a higher prevalence of irAEs during ipilimumab/nivolumab treatment and emphasizes that the higher frequency of irAEs is clinically relevant for the long‐term QoL of these patients. Other differences included in the presence of emotional fatigue, which was more common in ipilimumab/nivolumab, whereas single‐agent anti‐PD1 was more frequently associated with cognitive fatigue.

These results are comparable to findings of previous studies, indicating that melanoma survivors experience significant impairments in specific HRQL domains, including their physical and social role,^18^ emotional and neurocognitive functioning,^23^ as well as financial status.^24^ Fatigue was a common symptom in the present study, which is in line with previous reports and underlines its relevance in cancer survivors.^16^, ^17^, ^18^, ^23^, ^24^ Of note, the prevalence of fatigue differs across the different study reports, with frequency fluctuating between 28% and 90%.^16^, ^17^ This variability indicates a high heterogeneity in the reported symptom burden, although these results might be also affected by the disease stage, treatment modality and the different time points in the disease trajectory, including the time of study enrollment from the last ICI infusion. Approximately 50% of the patients in the present study were treated in the adjuvant setting. Consistent with previous reports,^16^ this study shows a correlation of endocrinological irAEs with fatigue, implying that specific patient populations might be in high‐risk for experiencing long‐term fatigue. Similarly, previous studies report a higher prevalence of fatigue in women^25^ and in elderly individuals.^26^ Such differences accentuate the importance of a personalized approach in survivorship care and emphasize the relevance of future studies in this field.

Analogous to previous reports,^16^ musculoskeletal symptoms were common after treatment discontinuation and were more prevalent in women, compared to men. The etiology of these long‐term musculoskeletal symptoms is multifactorial and might be in part explained by the frequency of irAEs during treatment. In a retrospective study investigating the prevalence of neuromuscular side effects in patients treated with ICIs, myositis was the most frequent irAE and it was either ongoing or had sequelae in approximately 50% of the patients after treatment discontinuation.^27^ In the current study, both ipilimumab/nivolumab and single‐agent anti‐PD1 were associated with musculoskeletal symptoms, which included myalgias and arthralgias, although muscle ache was numerically more evident in single‐agent anti‐PD1. Although there were no significant differences in the PRO‐CTCAE questionnaire between the two agents, it is well documented that anti‐PD1 treatment is more frequently associated with arthritis and myocarditis, whereas anti‐CTLA4‐driven toxicities include mostly colitis, hypophysitis, and skin rash.^28^

Previous studies indicate that psychosocial morbidity is common in melanoma patients. Despite the overall sustained HRQL, PROs demonstrate lower scores in psychosocial functioning, indicating increased emotional stress and neurocognitive impairment. Anxiety (31–72%) and especially fear of melanoma recurrence or disease progression have been previously reported in approximately 30–80% of the patients.^17^, ^18^, ^19^, ^23^, ^24^, ^29^, ^30^ In the present study, few patients reported anxiety and depression. Notably, 14% of the eligible candidates refused to participate in the study due to emotional distress. These results further argue the need for increased psychosocial support during cancer survivorship. Further knowledge on risk factors affecting patients' HRQL could aid in developing tailored survivorship care plans (SCPs).

We recognize existing study limitations, which include the single‐center study design, as well as the modest sample size (response rate to survey 68%), which also precludes from applying any multivariable models to analyze how the time on treatment and other confounders affect the reported results. Furthermore, although the study population only consisted of melanoma patients that received their last ICI infusion more than 6 months ago, there was heterogeneity in the time since treatment discontinuation, which prohibits drawing any conclusions on patients' symptoms at specific points in time during follow‐up.

The low number of missing data, the use of multiple validated questionnaires, and the long median time from treatment discontinuation to study enrollment are strengths of the present study. Additionally, these data provide more insight into the long‐term symptom burden and HRQL in melanoma survivors with results separated per treatment type.^16^, ^17^, ^18^ Besides, real‐life HRQL data investigating the long‐term symptoms in patients treated in the adjuvant setting are globally missing and this study adds to the current limited literature in this topic. As the number of melanoma survivors increase, it is crucial to review the frequency of chronic symptoms and persisting or new irAEs after treatment discontinuation to guide supportive care priorities and survivorship care. Despite the legitimate recognition of these toxicities in clinical trials, increasing evidence indicates that the reported rates of some irAEs might be in fact underestimated.^31^ It is therefore relevant in clinical practice to be conscious about the presence of long‐term symptoms that can potentially increase the patient's symptom burden and have an impact on their HRQL.

## 5 CONCLUSION

The advent of anti‐PD1‐based ICIs has significantly improved treatment outcomes in advanced melanoma with many patients experiencing long‐term disease control. With these innovative treatments, the number of patients living beyond treatment cessation is gradually increasing. Taking into account the increasing incidence of melanoma diagnosis, as well as the effective treatment options available, the number of melanoma survivors will continue to increase in the next few years. Cancer survivors, and particularly melanoma survivors, are being confronted with several issues, such as anxiety of disease recurrence, fertility issues, subsequent malignancies and long‐term side effects due to the previous systemic treatments. As such, there is an emerging need to address existing gaps in survivorship care and implement these findings in clinical practice, in order to improve patients' HRQL. These results suggest the importance to increase awareness for long‐term symptoms in melanoma survivors.

## AUTHOR CONTRIBUTIONS

**Esmée L. Looman:** Investigation (equal); project administration (equal); writing – original draft (equal). **Phil Cheng:** Formal analysis (lead). **Julia Lai‐Kwon:** Conceptualization (equal); methodology (equal). **Linda Morgan:** Investigation (equal). **Marliess Wakkee:** Investigation (equal). **Reinhard Dummer:** Resources (equal). **Florentia Dimitriou:** Conceptualization (equal); formal analysis (equal); investigation (equal); methodology (equal); supervision (lead); writing – original draft (equal); writing – review and editing (lead).

## FUNDING INFORMATION

This research did not receive any specific grant from funding agencies in the public, commercial, or not‐for‐profit sectors.

## CONFLICT OF INTEREST STATEMENT

FD receives/received honoraria and travel support from Merck Sharp & Dohme, Bristol Myers Squibb and Sun Pharma. RD declares intermittent, project focused consulting and/or advisory relationships with Novartis, Merck Sharp & Dhome (MSD), Bristol‐Myers Squibb (BMS), Roche, Amgen, Takeda, Pierre Fabre, Sun Pharma, Sanofi, Catalym, Second Genome, Regeneron, Alligator, T3 Pharma, MaxiVAX SA and touchIME outside the submitted work. All other authors have declared no conflicts of interest.

## ETHICS APPROVAL AND CONSENT TO PARTICIPATE

Ethics approval and consent to participate was obtained from the local ethics committee (KEK Zürich, BASEC‐Nr. 2022–00448). Patients' enrollment followed upon collection of written informed consent.

## Supporting information


Tables S1–S2.

Data S1.

## Data Availability

All data are available upon request.
