# Supplementary material for: Health‐related quality of life in survivors of advanced melanoma treated with anti‐PD1‐based immune checkpoint inhibitors
Source: Cancer Med. 2023 Apr 29;12(11):12861–73. doi: 10.1002/cam4.5967 (PMC10278493; doi:10.1002/cam4.5967)
Supplement: Supplementary file 1 — Tables S1–S2. [file CAM4-12-12861-s001.docx]

**Supplementary Table 1.** Immune-related adverse events (irAEs) leading to treatment discontinuation.

|  | Overall, N = 61 | anti-PD1, N = 40 | anti-PD1 and anti-CTLA4, N = 21 | p-value: 0.2^1^ |
| --- | --- | --- | --- | --- |
| Adrenal insufficiency | 1 (6%) | 1 (12%) | 0 |  |
| Arthritis | 3 (17%) | 1 (12%) | 2 (20%) |  |
| Colitis | 1 (6%) | 0 | 1 (10%) |  |
| Hepatitis | 1 (6%) | 0 | 1 (10%) |  |
| Hypophysitis | 1 (6%) | 0 | 1 (10%) |  |
| Myositis | 1 (6%) | 0 | 1 (10%) |  |
| Nephritis | 2 (11%) | 0 | 2 (20%) |  |
| Pancreatitis | 1 (6%) | 0 | 1 (10%) |  |
| Perimyocarditis | 1 (6%) | 1 (12%) | 0 |  |
| Pneumonitis | 3 (17%) | 3 (38%) | 0 |  |
| Polyneuropathy | 1 (6%) | 0 | 1 (10%) |  |
| Sarcoid-like immune reaction | 1 (6%) | 1 (12%) | 0 |  |
| Thrombocytopenia | 1 (6%) | 1 (12%) | 0 |  |

^1^Fisher's exact test

**Supplementary table 2.** The Cancer Related Fatigue Module (EORTC QLQ-FA12) in the overall study population and in patients with and without ongoing endocrine immune-related adverse events (irAEs)..

|  | Overall  N = 61 | No ongoing endocrine irAEs  N = 35 | With ongoing endocrine irAEs  N = 26 | p-value^1^ |
| --- | --- | --- | --- | --- |
| Have you lacked energy? | 14 (23%) | 5 (14%) | 9 (35%) | 0.062 |
| have you felt exhausted? | 13 (21%) | 3 (9%) | 10 (38%) | 0.005 |
| Have you felt slowed down? | 16 (26%) | 4 (11%) | 12 (46%) | 0.002 |
| Did you feel sleepy during the day? | 14 (23%) | 6 (17%) | 8 (31%) | 0.2 |
| Did you have trouble getting things started? | 15 (25%) | 6 (17%) | 9 (35%) | 0.12 |
| Did you feel discouraged? | 8 (13%) | 2 (6%) | 6 (23%) | 0.06 |
| Did you feel helpless? | 6 (10%) | 2 (6%) | 4 (15%) | 0.4 |
| Did you feel frustrated? | 7 (11%) | 4 (11%) | 3 (12%) | >0.9 |
| Did you have trouble thinking clearly? | 6 (10%) | 1 (3%) | 5 (19%) | 0.07 |
| Did you feel confused? | 3 (5%) | 1 (3%) | 2 (8%) | 0.6 |
| Did tiredness interfere with your daily activities? | 12 (20%) | 3 (9%) | 9 (35%) | 0.01 |
| Did you feel that your tiredness is (was) not understood by the people who are close to you? | 1 (2%) | 0 | 1 (4%) | 0.4 |

Results are collapsed: quite a bit/very much (3-4). Only these results are shown.

^1^Pearson's Chi-squared test; Fisher's exact test
