# Supplementary material for: Health‐related quality of life in survivors of advanced melanoma treated with anti‐PD1‐based immune checkpoint inhibitors
Source: Cancer Med. 2023 Apr 29;12(11):12861–73. doi: 10.1002/cam4.5967 (PMC10278493; doi:10.1002/cam4.5967)
Supplement: Supplementary file 2 — Data S1. [file CAM4-12-12861-s002.docx]

**NCI PRO-CTCAE ® ITEMS
Item Library Version 1.0
English
Form Created on 15 February 2022**

**As individuals go through treatment for their cancer they sometimes experience different symptoms and side effects. For each question, please select the one response that best describes your experiences over the past 7 days…**

| **1a.** In the last 7 days, what was the SEVERITY of your DRY MOUTH at its WORST? | | | | |
| --- | --- | --- | --- | --- |
| Ο None | Ο Mild | Ο Moderate | Ο Severe | Ο Very severe |

| **2a.** In the last 7 days, what was the SEVERITY of your DECREASED APPETITE at its WORST? | | | | |
| --- | --- | --- | --- | --- |
| Ο None | Ο Mild | Ο Moderate | Ο Severe | Ο Very severe |
| **2b.** In the last 7 days, how much did DECREASED APPETITE INTERFERE with your usual or daily activities? | | | | |
| Ο Not at all | ΟA little bit | Ο Somewhat | Ο Quite a bit | Ο Very much |

| **3a.** In the last 7 days, what was the SEVERITY of your DRY SKIN at its WORST? | | | | |
| --- | --- | --- | --- | --- |
| Ο None | Ο Mild | Ο Moderate | Ο Severe | Ο Very severe |

| **4a.** In the last 7 days, what was the SEVERITY of your ITCHY SKIN at its WORST? | | | | |
| --- | --- | --- | --- | --- |
| Ο None | Ο Mild | Ο Moderate | Ο Severe | Ο Very severe |

| **5a.** In the last 7 days, what was the SEVERITY of your NUMBNESS OR TINGLING IN YOUR HANDS OR FEET at its WORST? | | | | |
| --- | --- | --- | --- | --- |
| Ο None | Ο Mild | Ο Moderate | Ο Severe | Ο Very severe |
| **5b.** In the last 7 days, how much did NUMBNESS OR TINGLING IN YOUR HANDS OR FEET INTERFERE with your usual or daily activities? | | | | |
| Ο Not at all | Ο A little bit | Ο Somewhat | Ο Quite a bit | Ο Very much |

| **6a.** In the last 7 days, what was the SEVERITY of your PROBLEMS WITH CONCENTRATION at their WORST? | | | | |
| --- | --- | --- | --- | --- |
| Ο None | Ο Mild | Ο Moderate | Ο Severe | Ο Very severe |
| **6b.** In the last 7 days, how much did PROBLEMS WITH CONCENTRATION INTERFERE with your usual or daily activities? | | | | |
| Ο Not at all | Ο A little bit | Ο Somewhat | Ο Quite a bit | Ο Very much |

| **7a.** In the last 7 days, what was the SEVERITY of your PROBLEMS WITH MEMORY at their WORST? | | | | |
| --- | --- | --- | --- | --- |
| Ο None | Ο Mild | Ο Moderate | Ο Severe | Ο Very severe |
| **7b.** In the last 7 days, how much did PROBLEMS WITH MEMORY INTERFERE with your usual or daily activities? | | | | |
| Ο Not at all | Ο A little bit | Ο Somewhat | Ο Quite a bit | Ο Very much |

| **8a.** In the last 7 days, how OFTEN did you have PAIN? | | | | |
| --- | --- | --- | --- | --- |
| Ο Never | Ο Rarely | Ο Occasionally | Ο Frequently | Ο Almost constantly |
| **8b.** In the last 7 days, what was the SEVERITY of your PAIN at its WORST? | | | | |
| Ο None | Ο Mild | Ο Moderate | Ο Severe | Ο Very severe |
| **8c.** In the last 7 days, how much did PAIN INTERFERE with your usual or daily activities? | | | | |
| Ο Not at all | Ο A little bit | Ο Somewhat | Ο Quite a bit | Ο Very much |

| **9a.**  In the last 7 days, how OFTEN did you have a HEADACHE? | | | | |
| --- | --- | --- | --- | --- |
| Ο Never | Ο Rarely | Ο Occasionally | Ο Frequently | Ο Almost constantly |
| **9b.** In the last 7 days, what was the SEVERITY of your HEADACHE at its WORST? | | | | |
| Ο None | Ο Mild | Ο Moderate | Ο Severe | Ο Very severe |
| **9c.** In the last 7 days, how much did your HEADACHE INTERFERE with your usual or daily activities? | | | | |
| Ο Not at all | Ο A little bit | Ο Somewhat | Ο Quite a bit | Ο Very much |

| **10a.** In the last 7 days, how OFTEN did you have ACHING MUSCLES? | | | | |
| --- | --- | --- | --- | --- |
| Ο Never | Ο Rarely | Ο Occasionally | Ο Frequently | Ο Almost constantly |
| **10b.** In the last 7 days, what was the SEVERITY of your ACHING MUSCLES at their WORST? | | | | |
| Ο None | Ο Mild | Ο Moderate | Ο Severe | Ο Very severe |
| **10c.** In the last 7 days, how much did ACHING MUSCLES INTERFERE with your usual or daily activities? | | | | |
| Ο Not at all | Ο A little bit | Ο Somewhat | Ο Quite a bit | Ο Very much |

| **11a.** In the last 7 days, how OFTEN did you have ACHING JOINTS (SUCH AS ELBOWS, KNEES, SHOULDERS)? | | | | |
| --- | --- | --- | --- | --- |
| Ο Never | Ο Rarely | Ο Occasionally | Ο Frequently | Ο Almost constantly |
| **11b.** In the last 7 days, what was the SEVERITY of your ACHING JOINTS (SUCH AS ELBOWS, KNEES, SHOULDERS) at their WORST? | | | | |
| Ο None | Ο Mild | Ο Moderate | Ο Severe | Ο Very severe |
| **11c.** In the last 7 days, how much did ACHING JOINTS (SUCH AS ELBOWS, KNEES, SHOULDERS) INTERFERE with your usual or daily activities? | | | | |
| Ο Not at all | Ο A little bit | Ο Somewhat | Ο Quite a bit | Ο Very much |

| **12a.** In the last 7 days, what was the SEVERITY of your INSOMNIA (INCLUDING DIFFICULTY FALLING ASLEEP, STAYING ASLEEP, OR WAKING UP EARLY) at its WORST? | | | | |
| --- | --- | --- | --- | --- |
| Ο None | Ο Mild | Ο Moderate | Ο Severe | Ο Very severe |
| **12b.** In the last 7 days, how much did INSOMNIA (INCLUDING DIFFICULTY FALLING ASLEEP, STAYING ASLEEP, OR WAKING UP EARLY) INTERFERE with your usual or daily activities? | | | | |
| Ο Not at all | Ο A little bit | Ο Somewhat | Ο Quite a bit | Ο Very much |

| **13a.** In the last 7 days, what was the SEVERITY of your FATIGUE, TIREDNESS, OR LACK OF ENERGY at its WORST? | | | | |
| --- | --- | --- | --- | --- |
| Ο None | Ο Mild | Ο Moderate | Ο Severe | Ο Very severe |
| **13b.** In the last 7 days, how much did FATIGUE, TIREDNESS, OR LACK OF ENERGY INTERFERE with your usual or daily activities? | | | | |
| Ο Not at all | Ο A little bit | Ο Somewhat | Ο Quite a bit | Ο Very much |

| **14a.** In the last 7 days, how OFTEN did you feel ANXIETY? | | | | |
| --- | --- | --- | --- | --- |
| Ο Never | Ο Rarely | Ο Occasionally | Ο Frequently | Ο Almost constantly |
| **14b.** In the last 7 days, what was the SEVERITY of your ANXIETY at its WORST? | | | | |
| Ο None | Ο Mild | Ο Moderate | Ο Severe | Ο Very severe |
| **14c.** In the last 7 days, how much did ANXIETY INTERFERE with your usual or daily activities? | | | | |
| Ο Not at all | Ο A little bit | Ο Somewhat | Ο Quite a bit | Ο Very much |

| **15a.** In the last 7 days, how OFTEN did you FEEL THAT NOTHING COULD CHEER YOU UP? | | | | |
| --- | --- | --- | --- | --- |
| Ο Never | Ο Rarely | Ο Occasionally | Ο Frequently | Ο Almost constantly |
| **15b.** In the last 7 days, what was the SEVERITY of your FEELINGS THAT NOTHING COULD CHEER YOU UP at their WORST? | | | | |
| Ο None | Ο Mild | Ο Moderate | Ο Severe | Ο Very severe |
| **15c.** In the last 7 days, how much did FEELING THAT NOTHING COULD CHEER YOU UP INTERFERE with your usual or daily activities? | | | | |
| Ο Not at all | Ο A little bit | Ο Somewhat | Ο Quite a bit | Ο Very much |

| **16a.** In the last 7 days, how OFTEN did you have SAD OR UNHAPPY FEELINGS? | | | | |
| --- | --- | --- | --- | --- |
| Ο Never | Ο Rarely | Ο Occasionally | Ο Frequently | Ο Almost constantly |
| **16b.** In the last 7 days, what was the SEVERITY of your SAD OR UNHAPPY FEELINGS at their WORST? | | | | |
| Ο None | Ο Mild | Ο Moderate | Ο Severe | Ο Very severe |
| **16c.** In the last 7 days, how much did SAD OR UNHAPPY FEELINGS INTERFERE with your usual or daily activities? | | | | |
| Ο Not at all | Ο A little bit | Ο Somewhat | Ο Quite a bit | Ο Very much |

| **17a. In the last 7 days, what was the SEVERITY of your DECREASED SEXUAL INTEREST at its WORST?** | | | | | | |
| --- | --- | --- | --- | --- | --- | --- |
| Ο None | Ο Mild | Ο Moderate | Ο Severe | Ο Very severe | Ο Not sexually active | Ο Prefer not to answer |

| **OTHER SYMPTOMS** | | | | | | |
| --- | --- | --- | --- | --- | --- | --- |
| Do you have any other symptoms that you wish to report? | | | | | | |
| Ο Yes | | | Ο No | | | |
| **Please list any other symptoms:** | | | | | | |
| 1. | In the last 7 days, what was the SEVERITY of this symptom at its WORST? | | | | | |
|  | O None | O Mild | | O Moderate | O Severe | O Very Severe |
| 2. | In the last 7 days, what was the SEVERITY of this symptom at its WORST? | | | | | |
|  | O None | O Mild | | O Moderate | O Severe | O Very Severe |
| 3. | In the last 7 days, what was the SEVERITY of this symptom at its WORST? | | | | | |
|  | O None | O Mild | | O Moderate | O Severe | O Very Severe |
| 4. | In the last 7 days, what was the SEVERITY of this symptom at its WORST? | | | | | |
|  | O None | O Mild | | O Moderate | O Severe | O Very Severe |
| 5. | In the last 7 days, what was the SEVERITY of this symptom at its WORST? | | | | | |
|  | O None | O Mild | | O Moderate | O Severe | O Very Severe |
